# Processes of Obtaining Nanostructured Materialswith a Hierarchical Porous Structure on the Example of Alginate Aerogels

**DOI:** 10.3390/gels10120845

**Published:** 2024-12-20

**Authors:** Natalia Menshutina, Olga Fedotova, Andrey Abramov, Eldar Golubev, Yan Sulkhanov, Pavel Tsygankov

**Affiliations:** Department of Chemical and Pharmaceutical Engineering, Mendeleev University of Chemical Technology of Russia, Miusskaya pl. 9, 125047 Moscow, Russia; chemcom@muctr.ru (N.M.); abramovandrey516@gmail.com (A.A.); eldgol01@gmail.com (E.G.); yan.sulkhanov.dmi@gmail.com (Y.S.)

**Keywords:** hierarchical materials, hierarchical porous structures, aerogels, sodium alginate, supercritical drying

## Abstract

Currently, materials with specific, strictly defined functional properties are becoming increasingly important. A promising strategy for achieving these properties involves developing methods that facilitate the formation of hierarchical porous materials that combine micro-, meso-, and macropores in their structure. Macropores facilitate effective mass transfer of substances to the meso- and micropores, where further adsorption or reaction processes can occur. Aerogels represent a promising class of materials for implementing this approach. The formation of hierarchical porous structures in aerogels can be achieved using soft and hard templating methods or by foaming techniques. This paper presents a comprehensive study of three methods for forming hierarchical porous structures in alginate aerogels: (1) employing surfactants (Pluronic F-68), (2) using zein as a pore-forming component, and (3) foaming in a carbon dioxide medium. The results of micro-CT showed that each of the methods contributes to the formation of macropores within the structure of the resulting aerogels. Size distribution curves of the detected macropores were obtained, showing the presence of macropores ranging from 16 to 323 μm in size for samples obtained using surfactants, from 5 to 195 μm for samples obtained using zein, and from 20 μm to 3 mm for samples obtained by foaming in a carbon dioxide medium. The SEM images demonstrated the macro- and mesoporous fibrous structure of the obtained materials. The nitrogen porosimetry results indicated that samples obtained using surfactants and zein are characterized by a high specific surface area (592–673 m^2^/g), comparable to the specific surface area for an alginate-based aerogel obtained without the use of pore-forming components. However, the use of the developed methods for the formation of a hierarchical porous structure contributes to an increase in the specific mesopores volume (up to 17.7 cm^3^/g). The materials obtained by foaming in a carbon dioxide medium are characterized by lower specific surface areas (112–239 m^2^/g) and specific mesopores volumes (0.6–2.1 cm^3^/g). Thus, this paper presents a set of methods for forming hierarchical porous structures that can obtain delivery systems for active substances with a controlled release profile and highly efficient platforms for cell culturing.

## 1. Introduction

One of the most rapidly developing areas in materials science is the design of materials with hierarchical structures. Functional hierarchical materials are characterized by their structural organization at multiple levels, ranging from nanoscale to macroscale dimensions [1]. The properties of these materials are governed by both their composition and the spatial arrangement of their structural elements. Such materials exhibit unique mechanical, thermal, electrical, optical, and other functional properties, rendering them highly promising for various scientific and technological applications.

An especially compelling challenge is the development of technologies for fabricating hierarchical porous structures consisting of interconnected pores of diverse sizes [2]. According to the classification established by the International Union of Pure and Applied Chemistry (IUPAC) in 1972 [3], pores are categorized by size into micropores (diameters less than 2 nm), mesopores (diameters between 2 and 50 nm), and macropores (diameters exceeding 50 nm). Materials that incorporate two or three types of pores are considered to possess a hierarchical porous structure. The principal characteristic of such materials is that larger macropores facilitate rapid and efficient transport of substances to meso- and micropores, where subsequent adsorption or reaction processes occur.

Hierarchical porous structures can be precisely engineered for specific applications by adjusting pore size, shape, and distribution. This adaptability facilitates the development of materials with tailored properties for a wide range of applications, including energy conversion and storage [4], catalysis [5], and purification and filtration [6], as well as applications in biomedicine and pharmaceuticals [7].

The fabrication of hierarchical porous architectures can be accomplished by integrating techniques for hierarchical structure formation with supercritical drying technology. Through supercritical drying, the integrity of both micro- and mesoporous networks within the material is preserved [8]. Aerogels produced using supercritical drying possess a high specific surface area (over 200 m^2^/g), low density (0.003–0.2 g/cm^3^), and high porosity (at least 90%) [9]. Biopolymer-based aerogels have particular potential for creating effective therapeutic agents, for example, as delivery systems for active pharmaceutical ingredients (APIs) [10,11]. The high specific surface area enables a high loading of APIs, while the micro- and mesoporous structure facilitates the incorporation of APIs in an amorphous state, which, in turn, increases the bioavailability of the drug [10]. Moreover, aerogels can be used as a three-dimensional matrix that promotes efficient cell proliferation in organs and tissues [12,13,14]. However, cell sizes range from 10 to 100 μm. Accordingly, to ensure effective penetration and colonization of cells, the presence of macropores with a size of at least 10 μm is necessary, which can be achieved using methods for creating hierarchical porous structures.

**Figure 1:**
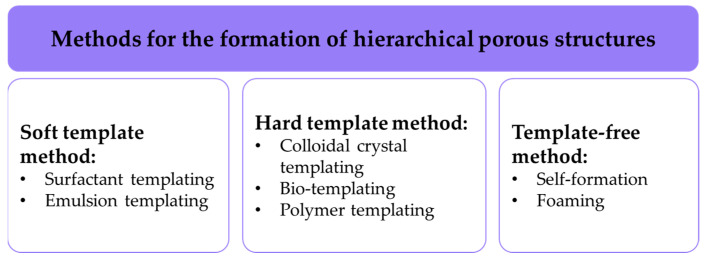
Methods for the formation of hierarchical porous structures.

Numerous methods for forming hierarchical porous structures are described in the scientific and technical literature; they can be broadly divided into three categories [4]: soft template methods, hard template methods, and template-free methods (Figure 1).

Template-based methods for fabricating hierarchical porous structures rely on the introduction of a prepared template that facilitates the formation of pores of specific sizes, followed by its subsequent removal. For example, surfactants or emulsions can be employed as soft templates. In Ref. [15], cellulose aerogels were obtained by freeze-drying Pickering emulsions. The resulting material was characterized by macropore sizes from 32–69 μm, a specific surface area of 45.6–148.3 m^2^/g, and a porosity of more than 99%. Another study [16] described a method for obtaining scaffolds based on sodium alginate using various types of nonionic surfactants. The macropore sizes for all the obtained materials were in the range of 50–300 μm, which ensured the proliferation of mesenchymal stem cells for 14 days. The authors of the study in [17] obtained alginate-based materials with a hierarchical porous structure using a surfactant. The obtained materials possessed an interconnected three-dimensional porous architecture, with a pore size in the range of 100–400 μm.

Hard templates, such as colloidal crystals, proteins, or polymers, can also be used. For example, in Ref. [18] the authors investigated the production of hollow spherical silicon particles using polystyrene as a template. Silica gel was applied to 200–250 nm polystyrene particles using tetraethoxysilane as a precursor and cetyltrimethylammonium bromide as a crosslinking agent. The template was removed by calcination at 550 °C. At various production parameters, the hollow particles had dimensions of 170–230 nm with a pore size of 2.2–50 nm. Another example involves using the biocompatible protein zein as a template. In Refs. [19,20] the process of obtaining starch-based aerogels using zein as a template for macropore formation was studied. The removal of the zein from the structure was achieved by dissolving it in ethyl alcohol. The results demonstrated that incorporating zein into the aerogel production process facilitated the formation of macropores (approximately 2 microns), even in compositions with a low zein content. Additionally, supercritical drying effectively preserved the material’s mesoporous structure.

Examples of template-free methods for forming hierarchical porosity are the methods of self-assembly and foaming in a medium of various gases. The authors of the work in [21] obtained a carbon aerogel with a hierarchical porous structure by freeze-drying of alginate gel followed by carbonization at 800 °C. The sizes of the macropores of the obtained aerogels were ~200–300 nm, and nitrogen porosimetry showed the presence of mesopores measuring 2–4 nm. In [22], a double syringe technique was proposed, which consisted in mixing a sodium alginate solution containing CaCO_3_ particles with a CO_2_/air gas mixture by introducing a gas/liquid mixture from one syringe into another. In this process, foaming and a decrease in the pH of the alginate solution were simultaneously carried out, leading to gelation. The process of gelation and foaming by the introduction of carbon dioxide can be carried out in high-pressure apparatus [23,24]. The pore size can be controlled by adjusting the pressure release rate [25].

Thus, template-based methods for forming hierarchical porous structures enable the creation of complex, multi-layered architectures with precise control over pore size and shape. In contrast, non-template methods are more cost effective. However, their major drawback is the limited ability to precisely control the morphology of the resulting structures.

This paper presents a comprehensive study of methods for fabricating hierarchical porous structures in alginate-based aerogels. Sodium alginate is one of the most widely used substances for producing biodegradable, highly porous materials [26]. Creating hierarchical porous structures in alginate-based materials enables the development of systems for delivering active substances—such as active pharmaceutical ingredients, growth factors, and biologically active additives—with controlled release profiles, as well as highly efficient platforms for cell cultivation. Three methods for macropore formation were investigated: employing surfactants, utilizing zein as a pore-forming agent, and foaming in a carbon dioxide environment. The micro- and mesoporous structures of the materials were preserved using supercritical drying technology. Analyzing the structural characteristics of the resulting aerogels provides insights into the effects of these methods on hierarchical porous structure formation.

## 2. Results and Discussion

This study investigated three methods for fabricating hierarchical porous structures of alginate-based aerogels:

1. Using surfactant (soft-template method);
2. Using zein as pore-forming agent (hard-template method);
3. Using a carbon dioxide medium (template-free method).

In the surfactant-based method, macropore formation is achieved via the gelation of a foamed sodium alginate solution. To produce the alginate foam, Pluronic F-68, a nonionic surfactant, is dispersed in the sodium alginate solution. However, the experimental results revealed that the resulting foam is unstable. Therefore, this study proposes the use of additional pore-forming agents: sodium bicarbonate, added to the sodium alginate solution, and acetic acid, incorporated into the crosslinking agent solution. During the gelation process, when the polymer solution containing sodium bicarbonate is introduced into the crosslinking agent solution containing acetic acid, a reaction occurs that releases carbon dioxide (1):

(1) $$NaHCO_{3}+CH_{3}COOH\to CH_{3}COONa+CO_{2}↑+H_{2}O$$

Gas generation enhances the intensity of foam formation, with the surfactant acting as a stabilizer for the resulting foam. The addition of the surfactant promotes the formation of adsorption layers on bubble surfaces, reducing surface tension at the liquid–gas interface. Consequently, this reduction in interfacial surface tension enhances the stability of air bubbles formed within the structure of sodium alginate-based particles.

In the method utilizing zein as a pore-forming agent, the formation of hierarchical porous structures involves dispersing zein particles in a sodium alginate solution. During the gelation process, structures with zein particles uniformly distributed throughout the volume are formed. Macropores are generated by dissolving the zein particles in alcohol during the subsequent solvent exchange stage.

The foaming method in a carbon dioxide medium is a template-free approach. Gelation of the sodium alginate solution is achieved through the introduction of CO_2_ under pressure (CO_2_-induced gelation). As pressure increases, the solubility of CO_2_ in water rises [27], leading to the formation of carbonic acid. This process leads to a decrease in the pH of the medium, facilitating the dissolution of calcium carbonate and inducing the gelation of alginate. Upon pressure release, CO_2_ transitions to its gaseous state, causing the material to foam and resulting in the formation of macropores. A distinctive advantage of this method is its ability to integrate the aerogel production stages within a single apparatus [23].

**Figure 2:**
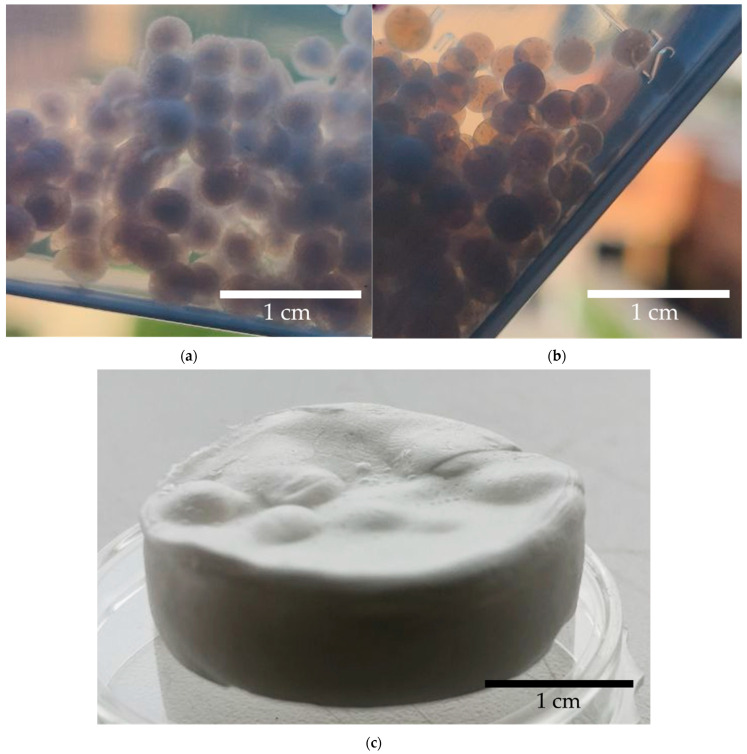
Appearance of samples obtained by (**a**)—using surfactants at a concentration of 0.5 wt.%; (**b**)—using zein with a sodium alginate–zein ratio of 5:1; (**c**)—using the template-free method by foaming in a carbon dioxide medium (calcium carbonate concentration 2 wt.%, pressure 100 bar, duration 3 h).

Figure 2 shows the appearance of the obtained samples.

**Figure 3:**
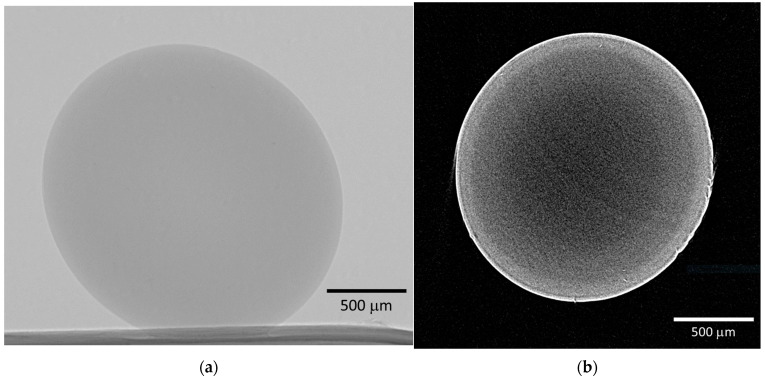
Micro-CT results of the reference sample: (**a**) shadow projection; (**b**) two-dimensional cross-section.

The macroporous structure of the samples was analyzed using X-ray microtomography. Figure 3 presents the shadow projection and a two-dimensional cross-section of the reference sample. The reference sample was prepared using the following procedure: preparation of a sodium alginate solution, gelation via dropwise introduction into a crosslinking agent solution (1 wt.% CaCl_2_), stepwise solvent exchange with isopropyl alcohol, and supercritical drying. The presented images demonstrate the absence of macropores larger than 2 μm in the samples, which is attributed to the resolution limit of the method.

**Figure 4:**
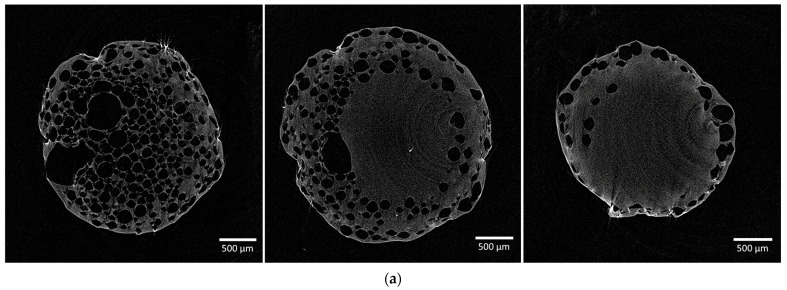

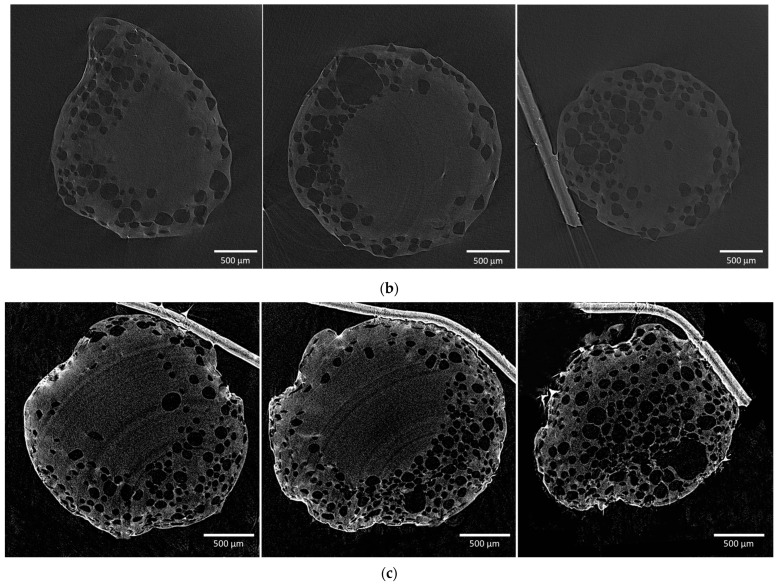
Micro-CT two-dimensional cross-sections in three planes of samples obtained using (**a**) 0.25 wt.% surfactant; (**b**) 0.50 wt.% surfactant; (**c**) 1.00 wt.% surfactant.

Figure 4 presents the micro-CT results for aerogel particles obtained using surfactant. Two-dimensional cross-sections in three planes reveal a well-developed macroporous structure in the outer shell of the particles, while the inner portion consists of a mesoporous structure. This material configuration arises from gas bubble formation at the interface where the polymer solution contacts the crosslinking agent solution on the droplet’s surface.

**Figure 5:**
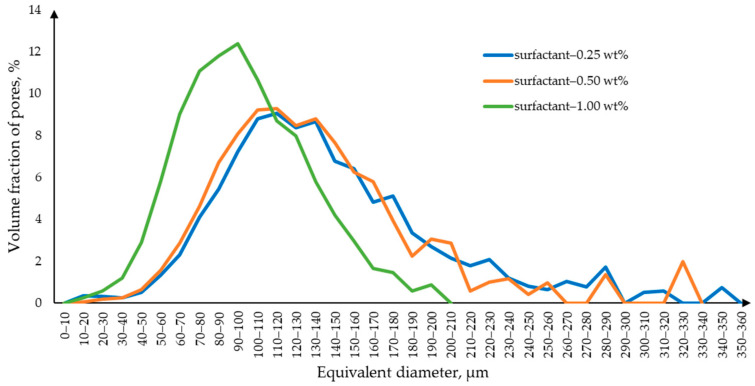
Macropore size distribution curve for samples obtained using surfactants.

Micro-CT data processing yielded pore size distribution curves (Figure 5), which show that with an increase in the surfactant concentration, the size of the macropores decreases. This is due to the fact that with an increase in the surfactant concentration, the stability of the foam increases, which helps to avoid bubble coalescence.

**Figure 6:**
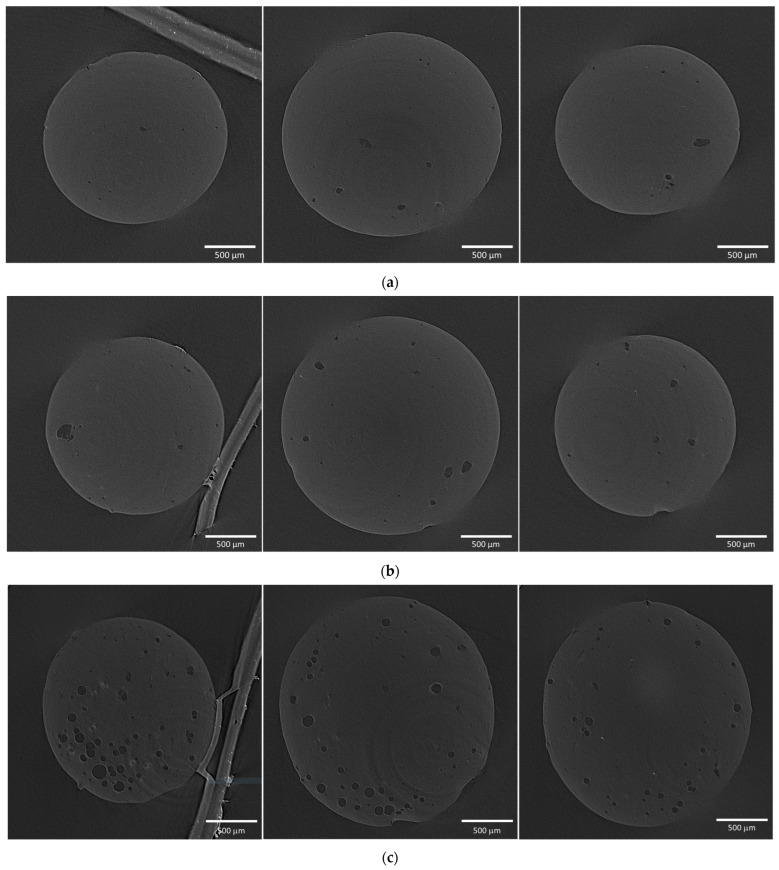
Micro-CT two-dimensional cross-sections in three planes of samples obtained using zein with a sodium alginate–zein ratio of (**a**) 20:1; (**b**) 10:1; (**c**) 5:1.

Figure 6 presents the micro-CT results for an aerogel particle obtained using zein as a pore-forming agent. The two-dimensional cross-sections reveal unevenly distributed macropores within the structure.

**Figure 7:**
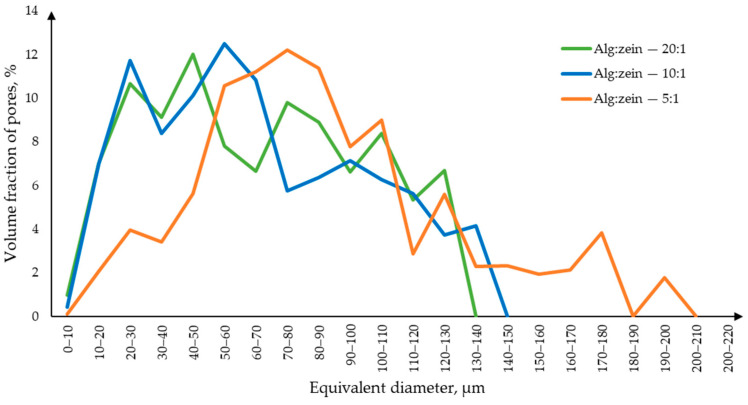
Macropore size distribution curve for samples obtained using zein.

The macropore size distribution curve (Figure 7) shows that samples with an alginate–zein ratio of 5:1 are characterized by larger macropores. This suggests that an increase in zein content leads to its agglomeration and, accordingly, to the formation of larger macropores.

**Figure 8:**
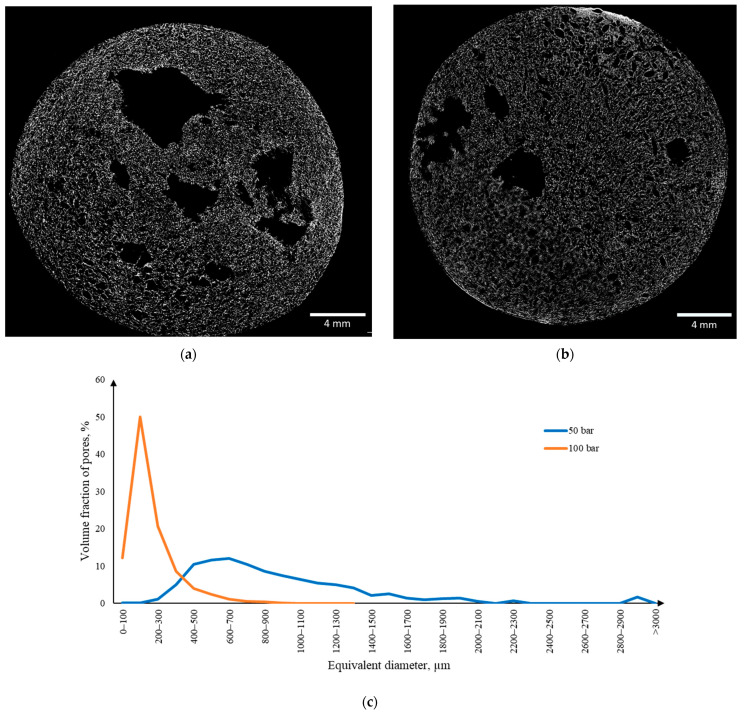
Micro-CT results of the sample obtained using the template-free method by foaming in a carbon dioxide medium: (**a**) two-dimensional cross-sections obtained at 50 bar pressure for 3 h; (**b**) two-dimensional cross-sections obtained at 100 bar pressure for 3 h; (**c**) differential macropore size distribution curve.

Figure 8 presents the micro-CT results for samples obtained using the template-free method by foaming in a carbon dioxide medium. Two-dimensional cross-sections of aerogels obtained at 50 bar pressure (Figure 8a) and at 100 bar pressure (Figure 8b) for 3 h are shown, demonstrating the presence of a developed macroporous structure. For the sample obtained at 100 bar pressure, the macropore size distribution curve is narrower (Figure 8c) compared to the sample obtained at 50 bar pressure. The macropore sizes for the sample obtained at 50 bar range from 20 μm to 3 mm, with the macropore volume accounting for 42.0% of the total sample volume. For the sample obtained at 100 bar, the macropore sizes range from 20 μm to 950 μm, with the macropore volume fraction being 28.5%. As the pressure increases, the solubility of carbon dioxide in water also rises. This, in turn, contributes to the formation of a denser structure and prevents the coalescence of bubbles that occur during depressurization.

Table 1 presents the results obtained from micro-CT data analysis, where D is the equivalent diameter and φ is the volume fraction of macropores relative to the total volume of the sample. The materials obtained using surfactants and foaming in a carbon dioxide environment are characterized by the most pronounced macroporous structure. For the samples produced using zein, the sizes of the macropores primarily depend on the particle sizes, which do not exceed 200 μm. The volume fraction of macropores relative to the total volume of the sample for this method is lower compared to other methods. Increasing the zein content to enhance the proportion of macropores is not feasible due to constraints associated with the methodology (Section 4.2.2).

**Figure 9:**
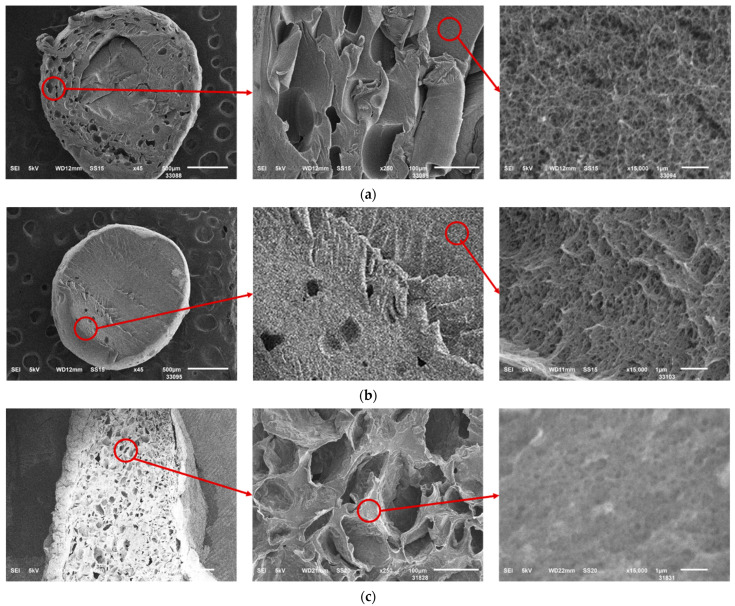
SEM images of the samples obtained by different methods: (**a**) using surfactants at a concentration of 0.5 wt.%; (**b**) using zein with a sodium alginate–zein ratio of 5:1; (**c**) using the template-free method by foaming in a carbon dioxide medium (calcium carbonate concentration 2 wt.%, pressure 100 bar, duration 3 h). The circle shows the increase in zoom.

Figure 9 presents scanning electron microscopy (SEM) images illustrate the macroporous structure of the samples obtained using the three methods. These images clearly demonstrate the presence of macropores within the material’s structure.

The schemes at ×45 and ×250 confirm the presence of macropores, while those at ×15,000 reveal the nanofibrous structure of the studied materials. Based on these observations, it can be concluded that all three methods enable the fabrication of aerogels with a hierarchical porous structure.

**Figure 10:**
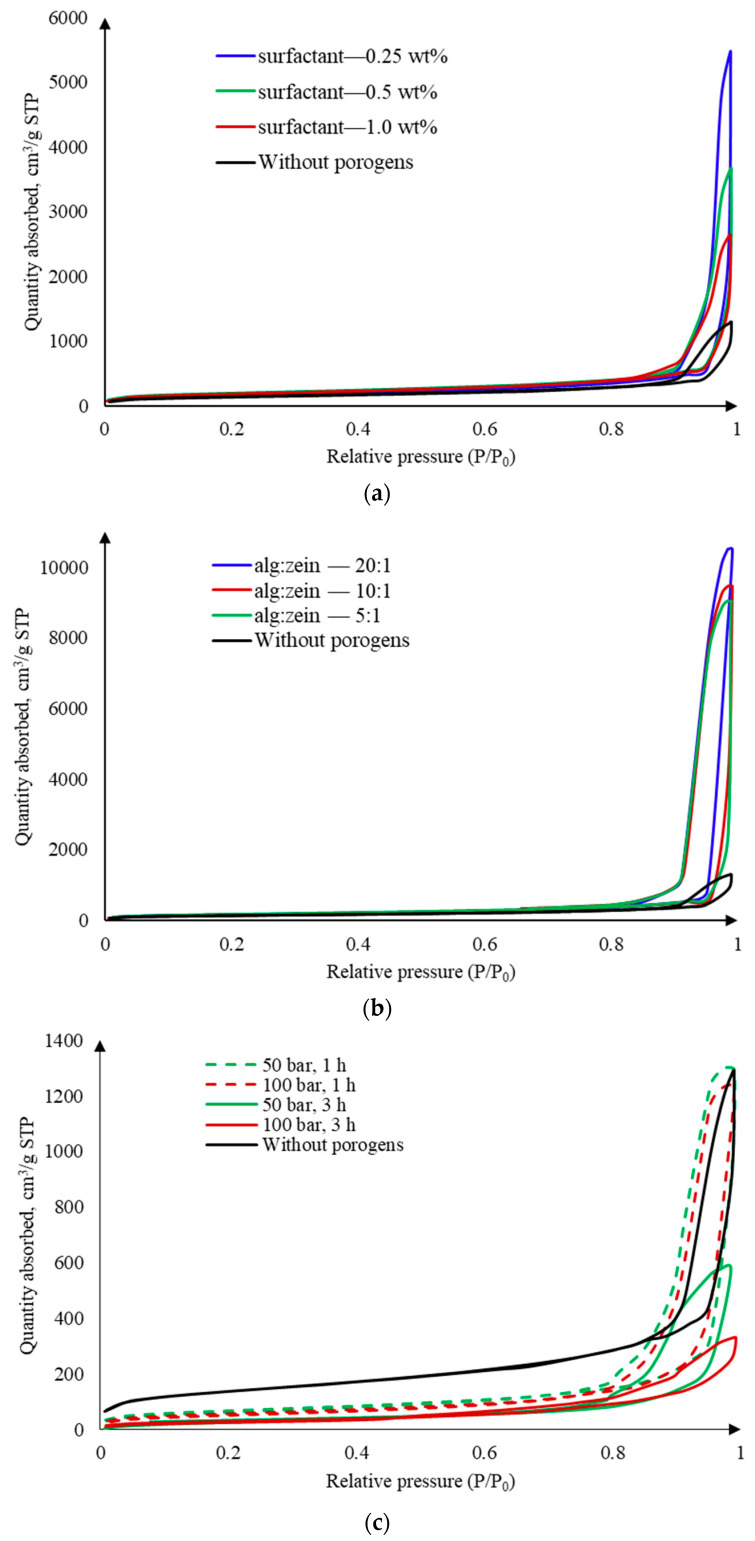
Nitrogen adsorption–desorption isotherms at 77 K for the samples obtained by different methods: (**a**) using surfactant; (**b**) using zein; (**c**) using template-free method by foaming in a carbon dioxide medium.

The mesoporous structure of the resulting aerogels was analyzed using nitrogen physisorption. The nitrogen adsorption–desorption isotherms at 77 K are shown in Figure 10.

According to the IUPAC classification [28], the adsorption–desorption isotherms obtained for all samples correspond to type IV. This type of isotherm is characteristic of reversible adsorption on mesoporous materials via a polymolecular adsorption mechanism. The isotherms exhibit characteristic hysteresis loops, indicating the occurrence of capillary condensation. For samples obtained using surfactants and zein as pore-forming agents (Figure 10a,b), the initial section of the isotherm (up to P/P_0_~0.8) coincides with that of the sample obtained without pore-forming components. In this range, the presence of micropores in the sample contributes significantly. However, the overall amount of adsorbed nitrogen increases substantially with the addition of a pore-forming agent, indicating an increase in mesopore volume. Thus, these template methods promote the formation of both macropores and a more developed mesoporous structure. For samples obtained using the foaming method in a carbon dioxide medium, the initial section of the isotherm lies below that of the sample obtained without pore-forming agents. This indicates a lower volume of micropores. Additionally, the total amount of adsorbed nitrogen is lower than in the sample without pore-forming components. Therefore, the foaming method in a carbon dioxide medium does not contribute to an increase in mesopore volume.

Table 2 presents the specific surface area $S_{BET}$, determined using the Brunauer–Emmett–Teller method (BET) and the specific mesopore volume $V_{BJH}$, determined using the Barrett–Joyner–Halenda method (BJH).

For samples obtained using surfactants and zein as a pore-forming agent, the specific surface area varies $S_{BET}$ slightly depending on the change in parameters. In addition, it is comparable to the specific surface area of the reference sample. According to the BET method, the specific surface area is determined at a limited range of relative pressures (0.05 < P/P_0_ < 0.3), which is characterized by filling of the monolayer. Thus, this fact also confirms the presence of micropores in these samples. It can be seen from the data obtained that for samples obtained using surfactants and zein as a pore-forming component, the specific volume of mesopores $V_{BJH}$ is higher than for the comparison sample, and to a much greater extent for samples obtained with the addition of zein. An increase in the concentration of surfactants and zein content leads to a decrease in the volume of mesopores due to the formation of structures with a large number of macropores. The samples obtained by foaming are characterized by a much lower specific surface area and mesopore volume. In addition, these values decrease with increasing pressure.

## 3. Conclusions

Hierarchical porous materials hold immense potential for applications across various fields, including pharmaceuticals and biomedicine. The development of hierarchical porous structures in aerogels can facilitate the creation of novel systems for controlled drug delivery and highly efficient platforms for cell cultivation. This study presents a comprehensive approach to fabricating hierarchical porous structures in alginate-based aerogels.

The first method—using surfactants (Pluronic F-68 in this study)—enables the creation of structures with spatial pore hierarchy: the outer shell of aerogel particles consists of macropores, while the inner part is mesoporous. The micro-CT results revealed macropore sizes ranging from 16 to 323 μm. The mesoporous structure of the samples is characterized by a high specific surface area (657–673 m^2^/g) and a high specific mesopore volume (4.0–8.6 cm^3^/g).

The second method—using zein as a pore-forming agent—produces structures with unevenly distributed macropores ranging from 5 to 195 μm in size. This method significantly increases the mesopore volume (15.1–17.7 cm^3^/g) and forms a mesoporous structure with a high specific surface area (592–640 m^2^/g).

The third method—foaming in a carbon dioxide medium—achieves macropores ranging from 20 μm to 3 mm without the use of pore-forming agents. The mesoporous structure of the resulting samples is characterized by a specific surface area of 112–239 m^2^/g. A key advantage of this method lies in the possibility of integrating aerogel production steps within a single apparatus.

Based on these results, all methods contribute to the formation of hierarchical porous structures in aerogels. The surfactant method yields a broad macropore size distribution and a well-developed mesoporous structure, with macropores located in the outer shell and mesopores concentrated within the internal structure. The zein method promotes the development of a highly mesoporous structure with significantly increased mesopore volumes, although it results in smaller macropores and lower macropore volumes. The CO_2_ foaming method produces a developed macroporous structure and is characterized by the highest macropore volume fraction among the studied methods. It is hypothesized that approaches employing surfactants and foam generation under carbon dioxide conditions may be utilized to establish cell culture platforms, in which mesopores facilitate the substantial loading of growth factors and biologically active agents, while macropores provide adequate space for cellular proliferation. Similarly, methodologies incorporating surfactants and the utilization of zein may be applied to the development of delivery systems for active substances, since the elevated specific surface area and mesoporous volume support the high-capacity loading of amorphous pharmaceutical compounds, and the presence of macropores ensures effective mass transport and controlled release. These propositions remain theoretical and necessitate further experimental verification.

In conclusion, this study presents a comprehensive set of methods for fabricating hierarchical porous structures in alginate-based aerogels. It has been demonstrated that the choice of method allows for tailoring the characteristics of the resulting materials to meet specific objectives, including the development of controlled drug delivery systems and platforms for cell cultivation.

## 4. Materials and Methods

### 4.1. Materials

Sodium alginate (Sigma-Aldrich, St. Louis, MO, USA) was used as the base material for aerogel production. Zein (Sigma-Aldrich, St. Louis, MO, USA), Pluronic F-68 (Gibco, China, Beijing), and sodium bicarbonate (RusChem, Moscow, Russia) were utilized as agents for macropore formation. Calcium carbonate (RusChem, Moscow, Russia) was used to facilitate gelation under pressure. Calcium chloride dihydrate (RusChem, Moscow, Russia) was employed to prepare the crosslinking agent solution. Isopropyl alcohol (IPA, RusChem, Moscow, Russia) served as the solvent, and distilled water was produced in the laboratory.

### 4.2. Methods

**Figure 11:**
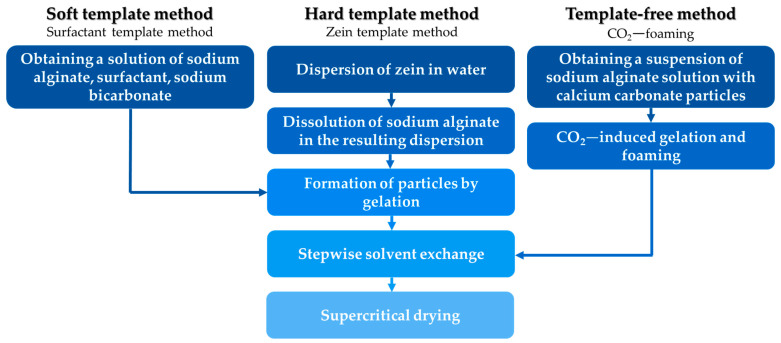
General scheme for producing alginate-based aerogels with a hierarchical porous structure.

This study explored three methods for forming hierarchical porous structures: the addition of surfactants, the addition of zein, and foaming in a carbon dioxide medium. Figure 11 presents the scheme for producing alginate-based aerogels with hierarchical porous structures using these methods.

Each of the methods is discussed in detail below.

#### 4.2.1. The Process of Obtaining Aerogel with a Hierarchical Porous Structure Using Surfactant

In the first stage, a solution consisting of sodium alginate, surfactant, and sodium bicarbonate is prepared. To ensure uniform distribution of sodium bicarbonate throughout the alginate solution, dispersion is carried out using a rotor–stator homogenizer IKA T 25 digital ULTRA-TURRAX (IKA-Werke, Germany, Staufen im Breisgau) at a rotor speed of 6000 rpm for 5 min. To study the effect of the initial solution composition on the particle structure, the surfactant concentration was varied from 0.25 to 1.00 wt.% while maintaining constant concentrations of sodium alginate (2 wt.%) and sodium bicarbonate (0.9 wt.%). The selection of surfactant concentration is based on the findings of preliminary experimental studies. A decrease in surfactant concentration results in the absence of stable alginate foam, which in turn leads to a reduction in the effectiveness of this pore-formation method.

The process of obtaining alginate particles involves introducing a polymer solution into a crosslinking agent solution using a syringe pump. Sodium alginate is an anionic polysaccharide copolymer of β-d-mannuronic acid and α-l-guluronic acid, which is capable of forming gels when reacting with divalent metal ions. In this method, a solution of acetic acid and calcium chloride with concentrations of 10 wt.% and 1 wt.%, respectively, was used as the crosslinking agent.

The resulting particles are held in the crosslinking agent solution for 24 h to complete the gelation process.

#### 4.2.2. The Process of Obtaining Aerogel with a Hierarchical Porous Structure Using Zein as Pore-Forming Agent

In the first stage, zein is dispersed in distilled water using a rotor–stator homogenizer. Sodium alginate is then dissolved in the resulting mixture. To achieve uniform distribution of zein throughout the solution, dispersion is carried out at a rotor speed of 6000 rpm for 5 min. The sodium alginate concentration in the solution is 2 wt.%, with alginate-to-zein ratios of 20:1, 10:1, and 5:1. For this method, it is not possible to consider a higher ratio, since a higher content of zein particles complicates the dripping process.

In the next stage, the prepared sodium alginate–zein suspension is introduced dropwise into a crosslinking agent solution using a syringe pump. The crosslinking agent is a pre-prepared aqueous solution of calcium chloride with a concentration of 1 wt.%. The resulting particles are held in the crosslinking agent solution for 24 h to complete the gelation process.

#### 4.2.3. The Process of Obtaining Aerogel with a Hierarchical Porous Structure Using Foaming in Carbon Dioxide Medium

In the first stage, an aqueous solution of sodium alginate with a concentration of 2 wt.% is prepared. Calcium carbonate particles are then added to the solution, and the resulting suspension is subjected to ultrasonic dispersion (Bandelin SONOPULS HD 4100, Berlin, Germany) at a frequency of 20 kHz and an amplitude of 30% for 5 min to achieve uniform distribution of the calcium carbonate particles.

After dispersion, the suspension is poured into Petri dishes and placed in a high-pressure apparatus with a 70 mL volume. The apparatus is sealed, and pressure is increased by filling it with liquid carbon dioxide. The pressure (50 and 100 bar) and the system’s holding time (1 and 3 h) are varied. The formation of a hierarchical porous structure in alginate materials in a carbon dioxide environment depends on the solubility of carbon dioxide in water and the diffusion of components within the volume of the materials. The range of pressures considered was selected based on the basis that the maximum operating pressure of the apparatus is 250 bar, and the solubility of carbon dioxide exhibits minimal variation between 100 and 250 bar [27]. Lower pressures were not considered due to the limited solubility of carbon dioxide under such conditions.

#### 4.2.4. Supercritical Drying

To perform the supercritical drying process, water in the gel pores is replaced with IPA in stages. A stepwise solvent exchange with increasing IPA content is conducted. Gradual alcohol concentration increases reduce gel shrinkage, as demonstrated in several studies [29,30]. The intervals between replacement stages are no less than 4 h. The final replacement stage (100% IPA) continues until the alcohol concentration reaches at least 98%.

Supercritical drying was carried out in accordance with the methodology presented in the work [31]. The supercritical drying process is carried out in a custom-designed high-pressure apparatus with a 250 mL volume. Before the process, the particles are packed in filter paper packages and placed in the apparatus. The apparatus is sealed, and liquid carbon dioxide is introduced. A piston pump raises the pressure to 120 bar, and the system is maintained for 20 min to establish equilibrium. During supercritical drying, the outlet valve is opened, and the carbon dioxide flow rate is set to 100 L/h under standard conditions. The process parameters are maintained constant: temperature 40 °C and pressure 120 bar. The process duration is 5 h. After drying, the carbon dioxide supply is stopped, and the pressure in the apparatus is released over 1 h. Once atmospheric pressure is reached, the apparatus is opened, and the packages with the dried samples are removed.

#### 4.2.5. Characterization

The samples were studied using computer tomography with a SkyScan-1172 microtomograph (Bruker Corporation, Karlsruhe, Germany). The following imaging parameters were applied during the investigation of the aerogel samples: X-ray source voltage/current 25–50 kV/100–118 µA, nominal resolution 1.49 µm, and a sample rotation angle of 0.2°.

The internal structure of the material was examined using scanning electron microscopy (SEM) on a JEOL 1610LV (JEOL, Japan, Tokyo) microscope at the Mendeleev Collective Use Center.

The mesoporous structure of the samples was investigated using low-temperature nitrogen adsorption (77 K) on an NOVA 2200E surface area and porosity analyzer (Quantachrome Instruments Corp., Boynton Beach, FL, USA). Before porosimetry, the samples were degassed at a pressure of 0.5 mm Hg and a temperature of 313 K for 12 h to remove all adsorbed moisture from the sample surfaces. The specific surface area was determined using the Brunauer–Emmett–Teller (BET) method, while pore size distribution and mesopore volume were calculated using the Barrett–Joyner–Halenda (BJH) method.

## Figures and Tables

**Table 1:** Micro-CT results.

Method	Parameter	D, µ	φ, %
Using surfactant	Surfactant 0.25	14-341	36.5
	Surfactant 0.50	16-323	22.5
	Surfactant 1.00	14-197	21.9
Using zein	Alg–zein—20:1	5-161	0.3
	Alg–zein—10:1	5-201	0.7
	Alg–zein—5:1	5-195	4.5
Foaming in carbon dioxide medium	50 bar, 3 h	20-3000	42.0
	100 bar, 3 h	20-950	28.5

**Table 2:** Results of nitrogen etry.

Method	Parameters	$S_{BET}$, m^2^/g	$V_{BJH}$, cm^3^/g
Reference sample	-	614	2.5
Using surfactant	Surfactant 0.25	609	8.6
	Surfactant 0.50	657	5.6
	Surfactant 1.00	673	4.0
Using zein	Alg–zein—20:1	592	17.7
	Alg–zein—10:1	617	15.8
	Alg–zein—5:1	640	15.1
Foaming in carbon dioxide medium	50 bar, 1 h	239	2.1
	100 bar, 1 h	127	0.9
	50 bar, 3 h	206	2.0
	100 bar, 3 h	112	0.6

## Data Availability

The original contributions presented in this study are included in the article. Further inquiries can be directed to the corresponding authors.
